# Analyses of a set of 128 ancestry informative single-nucleotide polymorphisms in a global set of 119 population samples

**DOI:** 10.1186/2041-2223-2-1

**Published:** 2011-01-05

**Authors:** Judith R Kidd, Françoise R Friedlaender, William C Speed, Andrew J Pakstis, Francisco M De La Vega, Kenneth K Kidd

**Affiliations:** 1 Department of Genetics, Yale University School of Medicine, 333 Cedar Street, New Haven, CT 06510, USA; 2 Independent researcher, Sharon, CT 06069, USA; 3 Life Technologies, Foster City, CA 94404, USA

## Abstract

**Background:**

Using DNA to determine an individual's ancestry from among human populations is generally interesting and useful for many purposes, including admixture mapping, controlling for population structure in disease or trait association studies and forensic ancestry inference. However, to estimate ancestry, including possible admixture within an individual, as well as heterogeneity within a group of individuals, allele frequencies are necessary for what are believed to be the contributing populations. For this purpose, panels of ancestry informative markers (AIMs) have been developed.

**Results:**

We are presenting our work on one such panel, composed of 128 ancestry informative single-nucleotide polymorphisms (AISNPs) already proposed in the literature. Compared to previous studies of these AISNPs, we have studied three times the number of individuals (4,871) in three times as many population samples (119). We have validated this panel for many ancestry assignment and admixture studies, especially those that were the rationale for the original selection of the 128 SNPs: African Americans and Mexican Americans. At the same time, the limitations of the panel for distinguishing ancestry and quantifying admixture among Eurasian populations are noted.

**Conclusion:**

We demonstrate the simultaneous importance of the specific set of population samples and their relative sample sizes in the use of the *structure *program to determine which groups cluster together and consequently influence the ability of a marker panel to infer ancestry. We demonstrate the strengths and weaknesses of this particular panel of AISNPs in a global context.

## Background

In recent years, there have been many proposed ancestry informative markers (AIMs) and published sets of AIMs useful for particular purposes. Some sets have focused on estimating the admixture between specific ancestral populations such as the African and European genetic contributions to African Americans or European, Native American and African contributions to Latino populations [e.g., [1-7]. Others have focused on distinguishing ancestral origins from three or four continental regions, such as sub-Saharan Africa, Europe, East Asia and the Americas [8-12], or more broadly between many globally distributed populations [13-16]. Yet others have focused on identifying the stratification of populations within particular geographic areas [e.g., [17-19] or within a clinical association study sample [20-22]. Whatever the purpose, the general usefulness of such AIMS depends very much on the set of populations used to identify and characterize them. Some global studies have used only a few but widely separated population samples [e.g., [15]. Others have used the HGDP-CEPH panel [23] of about 1,000 samples from 52 populations to select AIMs [10,11]. All approaches provide useful data but may also have weaknesses due to sampling error, either because the population samples used may not be highly representative of a broader geographic area or because the individual sample sizes are very small and subject to very large sampling errors. The same criticism applies to studies attempting to identify markers that provide ancestry information within a region, such as East Asia [24] or Europe [17,18].

Whatever the strategy for identifying them, AIMs are necessarily selected because they distinguish the specific population samples used. Therefore, replication with other samples of individuals from the same and/or closely related populations is necessary to verify the robustness of any set of AIMs. Such replication is onerous, costly and rarely undertaken.

Given the broad interest in AIMs in genetics, medicine, anthropology and forensics, the development of an optimal set of AIMs for a broad range of uses needs to be based on multiple markers studied on moderate to large samples of multiple relevant populations; appropriate resources will probably not be available in any single lab. As we advocated in the case of single-nucleotide polymorphisms (SNPs) for individual identification [25,26], multiple labs need to test candidate markers on additional populations and for general robustness in the laboratory. While very large numbers of markers can provide quite accurate ancestry information for multiple geographic regions, small but robust sets of markers are especially useful.

Seldin's group [6,27] identified a set of 128 SNPs that they showed is useful for identification of the continental origin of people and in estimating the admixture proportions of these individuals. Thus, a particular aim was to develop a set of SNPs whose allele frequencies had major differences between the continental populations for use in matching controls and subjects in association studies. They validated all and various subsets of the 128 SNPs in their initial study of 825 individuals from 20 designated populations and subsequently in a study of 1,620 individuals from 48 population samples using a subset of 93 of the 128 SNPs [27].

Understanding that a set of AIMs (or ancestry identification SNPs, AISNPs) will only be broadly useful for population relationships and for identifying admixture if that set can be shown on a very large data set to be valid, we have tripled the size of the Nassir *et al*. [27] population set, increased the number of population samples to 119 and analyzed this sample with the 128 AIMs of Kosoy *et al*. [6]. We find that this set of 128 AISNPs is not only globally informative for origins from major geographic regions but also informative for distinguishing relationships within several of those regions. This provides further support for the usefulness of this set of SNPs in some ancestry/admixture analyses. We also note that these AISNPs are not particularly good at distinguishing within certain groups of populations, and a comparison of the Nassir *et al*. [27] results with ours illustrates effects of choice and size of the population samples analyzed.

## Methods

### Samples

We assembled a data set of samples of 4,871 individuals: those from the HapMap 3 [28], the Human Genome Diversity Project (HGDP) [29,30] and our lab, all typed for the 128 SNPs of Kosoy *et al*. [6]. Some of the HGDP samples used by Nassir *et al*. [27] are also included in our study, and for some of their populations we have an independent sample, e.g., Ashkenazi Jews. The HGDP contains 355 DNA samples from our lab or from cell lines we hold and routinely type in our lab and another 31 HGDP DNA samples are DNA samples we also have in our lab. When an HGDP sample is a subset of one of our population samples, we used only our inclusive sample. When a sample from our lab overlapped with an HGDP sample, the duplicates were removed from our sample and the full HGDP sample was included separately from our supplementary sample. Thus, we occasionally have two samples from the same population (e.g., Druze, PNG, Makrani), but no individuals from the two samples overlap. Sixteen populations are represented by two to four samples. Some of the "duplicate" populations (e.g., Han, Russians, Maasai) were sampled in different areas or countries, and some of the "duplicate" populations are independent samples from the same locale. Finally, the offspring in the HapMap 3 samples (ASW, CEU, MKK, YRI, and MEX) were removed so that the samples include only unrelated people. Table 1 provides the name, sources of the data and sample size for each of the final set of 119 population samples. All samples from our lab were collected with informed consent under protocols approved by the Institutional Review Board (IRB) at Yale University and other relevant IRBs; the other data are in the public domain. Descriptions of all of the population samples are in ALFRED [28] associated with the allele frequencies.

**Table 1:** Name, source of data, and sample size for the 119 population samples*

Population	Abbreviation	N	Source
Biaka	BIA	67	Yale*
Mbuti	MBU	39	Yale*
Mandenka	MND	24	HGDP*
Lisongo	LSG	8	Yale
Yoruba	YOR	77	Yale
YorubaYRI	YRI	113	HapMap*
Ibo	IBO	48	Yale
Zaramo	ZRM	36	Yale
Hausa	HAS	39	Yale
Bantu_NE	BTN	12	HGDP*
Bantu_S	BTS	8	HGDP*
San	SAN	6	HGDP*
Luhya LWK	LWK	90	HapMap
African American 1	AAM	90	Yale
African American ASW	ASW	56	HapMap
Chagga	CGA	45	Yale
Maasai, T	MAS	20	Yale
Maasai MKK	MKK	144	HapMap
Sandawe	SND	40	Yale
Ethiopian Jews	ETH	32	Yale
Somali	SML	12	Yale
Mozabite	MOZ	30	HGDP*
Kuwaiti	KWT	16	Yale
Samaritans	SAM	40	Yale
Yemenite Jews	YMJ	42	Yale
Palestinian 1	PLA-1	49	Yale
Palestinian 2	PLA-2	51	HGDP*
Druze 1	DRU-1	75	Yale
Druze 2	DRU-2	47	HGDP*
Bedouin	BDN	48	HGDP*
Roman Jews	RMJ	26	Yale
Adygei	ADY	54	Yale*
Greeks	GRK	53	Yale
Ashkenazi Jews	ASH	79	Yale
Tuscan 1	Tus	8	HGDP
Tuscan TSI	TSI	88	Hapmap
Sardinian 1	SRD-1	34	Yale
Sardinian 2	SRD-2	28	HGDP
Orcadian	ORC	16	HGDP
North_Italian	ITN	13	HGDP
French_Basque	FRB	24	HGDP*
French	FRN	29	HGDP
Hungarians	HGR	89	Yale
Irish	IRI	114	Yale
European American 1	EAM	89	Yale
European Amer CEU	CEU	115	HapMap*
Russians 1	RUA	33	Yale
Russians 2	RUV	47	Yale*
Finns	FIN	34	Yale
Danes	DAN	51	Yale
Komi Zyriane	KMZ	47	Yale
Chuvash	CHV	42	Yale
Makrani 1	MKR-2	26	Yale
Makrani 2	MKR-1	25	HGDP
Kalash	KLS	25	HGDP*
Brahui	BRH	25	HGDP
Balochi	BCH	25	HGDP*
Sindhi	SDI	25	HGDP
Keralite	KER	30	Yale
Thoti	THT	14	Yale
Kachari	KCH	17	Yale
Gujarati GIH	GIH	88	HapMap
Pathan 1	PTH-1	75	Yale
Pathan 2	PTH-2	23	HGDP
Mohanna	MHN	48	HGDP
Burusho	BSH	25	HGDP*
Khanty	KTY	50	Yale
Hazara 1	HZR-1	87	Yale
Hazara 2	HZR-2	24	HGDP
Uygur 2	UYG	10	HGDP*
Uygur 1	UIG	45	Yale
Khazak	KAZ	44	Yale
Khamba Tibetan	KHG	27	Yale
Mongolians 1	MVF	62	Yale
Mongolians 2	MGL	10	HGDP*
HmongBlack	HMQ	46	Yale
BaimaDee	BQH	40	Yale
Qiang	QMR	38	Yale
Hlai	LIC	47	Yale
Yakut	YAK	51	Yale*
Dai	DAI	10	HGDP
Lahu	LHU	10	HGDP*
Miaozu	MIZ	10	HGDP
Naxi	NXI	9	HGDP
Oroqen	OQN	10	HGDP
She	SHE	10	HGDP
Tu	TU	10	HGDP
Tujia	TUJ	10	HGDP
Xibo	XBO	9	HGDP
Yizu	YIZ	10	HGDP
Daur	DUR	9	HGDP*
Hezhen	HEZ	9	HGDP
Han, SF	HAN	43	HGDP
Han CHD	CHD	85	HapMap
Han CHB	CHB	84	HapMap*
Han, Taiwan	CHT	50	Yale
Hakka	HKA	41	Yale
Koreans	KOR	54	Yale
Japanese	JPN	50	Yale
Japanese JPT	JPT	86	HapMap*
Laotians	LAO	118	Yale
Cambodians	CBD	24	Yale*
Ami	AMI	40	Yale
Atayal	ATL	42	Yale
Malaysians	MLY	11	Yale
Micronesians	MCR	34	Yale
Samoans	SMO	8	Yale
P-NG 1	PNG	13	Yale
P-NG 2	PNG	17	HGDP*
Nasioi	NAS	22	Yale
Mexican Amer MEX	MEX	49	HapMap*
Pima Mexico	PMM	53	Yale*
Maya	MAY	51	Yale*
Quechua	QUE	22	Yale
Colombians	COL-2	13	HGDP*
Guihiba	COL-1	11	Yale
Ticuna	TIC	65	Yale
Surui R	SUR	45	Yale
Karitiana	KAR	55	Yale

*These or subsets of these samples were included in Nassir *et al*. (2008). Descriptions of the populations and samples are in ALFRED.

### Marker Data

The polymorphic sites were those reported by Kosoy *et al*. [6]. The 3,071 samples from our lab were typed by TaqMan SNP Genotyping Assays^® ^(Applied Biosystems, Foster City, California, USA). The HGDP marker data were downloaded from http://hagsc.org/hgdp/files.html[31]. The HapMap data were downloaded from http://hapmap.ncbi.nlm.nih.gov/index.html.yo[28]. Of the 128 SNPs typed for 119 population samples, only 16 instances (one AISNP for one population) of missing data existed in the public data. Eleven SNPs in seven HapMap 3 populations do not have genotype data available, and our estimates for those frequencies do not significantly affect the PCA results (Additional File 1).

### Fst

Fst was calculated across all populations for each marker using the simple formula of Wright [32]: σ2pq¯. For comparison, Fst was calculated for 2,327 other polymorphisms typed on our samples. None of these 2,327 polymorphisms included sites specifically selected for admixture or ancestry identification, or for individual identification; instead, they were all selected for other ongoing projects in our lab (i.e., linkage disequilibrium, disease/disorder association).

### PCA

Principal component analysis (PCA) analyses of population sample allele frequencies were performed using XLSTAT (version 2009.4.07; Addinsoft SARL, http://www.xlstat.com/en/company/)) as one method to evaluate effectiveness of these SNPs for distinguishing among populations and to determine the major factors accounting for the population frequencies.

#### Structure

*Structure *(version 2.3.3; software freely available at http://pritch.bsd.uchicago.edu/structure.html[33-35]) was also used to evaluate and illustrate the effectiveness of these sites to distinguish among these populations. The burn-in was set at 20,000 followed by 10,000 iterations, and a model of correlated allele frequencies was specified. Ten replicates at each "K" levels 2-6 and 20 replicates at *K *= 7 and *K *= 8 were evaluated using CLUMPP; (software freely available at http://rosenberglab.bioinformatics.med.umich.edu/clumpp.html) [36]. Specific solutions have been plotted using DISTRUCT 1.1; software freely available at http://rosenberglab.bioinformatics.med.umich.edu/distruct.html) [37]. The matrix of pairwise similarities among replicate runs was used to identify different overall patterns based on high G values among runs with the "same" pattern and lower values for runs with different patterns.

## Results

### New data

The allele frequencies for the 128 AISNPs for all 119 population samples are given in Additional file 2, and the allele frequencies of the 69 population samples tested in our lab have all been entered into the ALFRED database [30] and can be readily accessed using the rs# of each SNP.

### Fst

**Figure 1:**
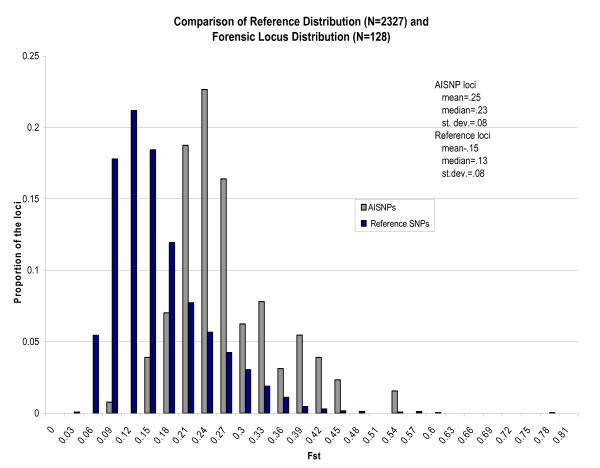
**Comparisons of Fst distributions for the 128 ancestry informative single-nucleotide polymorphisms (AISNPs) and for a reference set of 2327 SNPs**.

The Fst distribution of the 128 AISNPs was compared to the distribution of 2,327 non-AISNPs typed in our lab (Figure 1 and Additional file 3). Although Kosoy *et al*. [6] selected their 128 AISNPs not on the basis of Fst, but rather on the Informativeness statistic (I_n_) of Rosenberg *et al*. [38,39], Fst clearly separates the two distributions by 1.25 standard deviations. The null hypothesis that the two distributions are the same is rejected with a probability considerably less than 0.001. Outliers in the two distributions are given in Additional file 4. At the high-Fst end of the distributions, there are nine sites with Fst greater than 0.48: seven are in the reference distribution, and two are in the AISNP distribution. Of the seven in the reference distribution, five are located in or near genes of known phenotypic effect (*SLC24A5, OCA2 *(two SNPs), *HERC2 *and *EDAR*), and each of these genes is well known to have SNPs with marked global variation in allele frequency; but the best "known" SNPs are not part of this 128 AISNP set (Additional file 4). Though not associated with a phenotype, the remaining two "outliers" in the reference distribution have comparably high Fst values (Additional file 4). The two outliers at the high end of the AISNP distribution are sites in or near *EDAR *(rs260690, Fst = 0.5205) and *RTTN *(rs4891825, Fst = 0.5176). There are 10 outliers at the low end of the reference Fst distribution with Fst <0.04. Only one of the AISNPs falls below the mode of the reference distribution: *TWGS1 *(rs4798812, Fst = 0.08753).

### PCA

**Figure 2:**
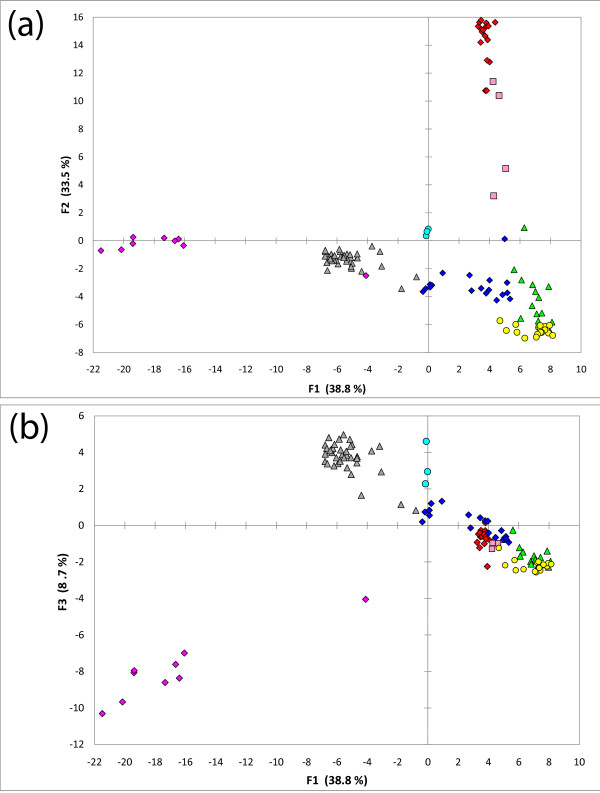
**Principal component analysis (PCA) of 119 population samples based on allele frequencies of 128 AISNPs**.

Figure 2 presents the first three factors of the PCA analysis based on allele frequencies of each of the 119 samples. The first two factors account for more than 72% of the variance. Factor 3 accounts for an additional 8.7% of the total variance. Factor 1 clearly separates the Native Americans from all other groups, and factor 2 clearly separates the African populations from the rest. Factor 3 emphasizes the difference between Native Americans and East Asians. This set of AISNPs was selected by Kosoy *et al*. [6] to maximize the differences among European Americans, Africans and Native Americans; those three groups clearly are at the vertices of the triangular pattern based on factors 1 and 2 (Figure 2). We also note that Eurasian populations show less clear separation.

#### Structure

**Figure 3:**
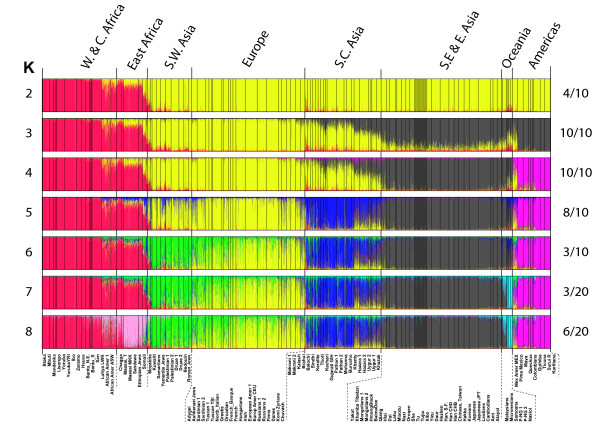
***Structure *analyses at *K *= 2-8 for 119 population samples**.

**Figure 4:**
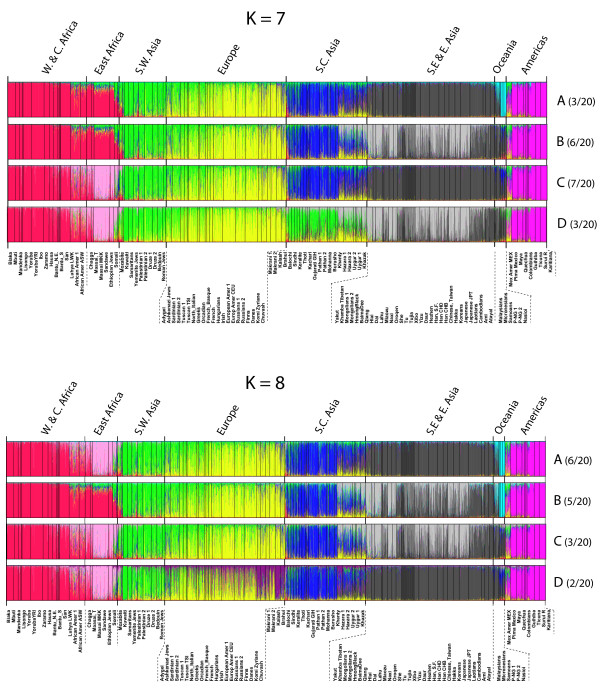
**The different patterns seen more than once in solutions from 20 runs of *structure *at *K *= 7 and *K *= 8**.

Results for the specific *structure *runs with the highest likelihood at each *K *value, *K *= 2-8, are shown in Figure 3 along with the number of times the particular overall pattern occurred. At all the *K *values, there are populations in each of the groups that seem quite homogeneous. By *K *= 8, the likelihoods began to plateau (Additional file 5), providing a statistically reasonable stopping point. For a better understanding of the ability of the data to distinguish most likely ancestry at the higher *K *values, we ran *structure *a total of 20 times, and the patterns seen more than once are illustrated in Figure 4; the patterns and likelihoods of the individual runs are given in Table 2. As is obvious from the patterns and the likelihoods of the individual runs, some distinctions are quite consistent while others generate similar likelihoods with combinatorial alternatives for a few different groups of populations.

**Table 2:** Patterns and likelihoods of 20 structure runs at K = 7 and K = 8

Pattern *K *= 7	LnP(D)	Run	Best per pattern	Pattern *K *= 8	LnP(D)	Run	Best per pattern
A	-591354	run13	*	A	-590090	run13	*
B	-591528	run1	*	B	-590185	run1	*
B	-591555	run2		B	-590570	run6	
A	-591571	run3		B	-590605	run16	
B	-591707	run12		A	-590606	run15	
B	-591724	run8		C	-590867	run4	*
C	-591822	run7	*	A	-591033	run18	
A	-591855	run17		A	-591053	run2	
B	-591944	run15		C	-591080	run20	
C	-591949	run5		A	-591090	run10	
C	-591957	run9		E	-591160	run5	
C	-592012	run11		C	-591298	run14	
B	-592017	run4		C	-591371	run3	
D	-592137	run20	*	D	-591512	run7	*
D	-592272	run18		D	-591689	run17	
E	-592309	run6		A	-591744	run12	
C	-592342	run16		C	-591745	run8	
C	-592548	run19		F	-592008	run19	
C	-592605	run14		G	-592162	run11	
D	-593102	run10		H	-592261	run9	

At *K *= 7, there is no single solution clearly identifiable as best. Five different overall patterns occur in the 20 runs. The pattern illustrated in Figure 3 has the highest likelihood but is not the most common pattern. The next highest likelihood is nearly identical and occurs for a pattern that occurs in 6 of the 20 runs and differs by subdividing East Asian populations and not distinguishing the Pacific populations. The most frequent pattern, found for 7 of the 20 runs, does not have the highest likelihoods and differs in separating East African populations from the Pacific populations.

At *K *= 8, eight different overall patterns occur among the 20 separate runs. The pattern shown is the most commonly found and does have the highest likelihood among the 20 runs. A nearly equal likelihood occurred for a somewhat different overall pattern that subdivides East Asia rather than separating East African populations.

At K = 8, the results can be summarized with respect to the pattern shown in Figure 3 (pattern A in Figure 4). Starting from the left, the sub-Saharan Africans, especially the West Africans, seem relatively homogeneous (red). The East Africans, especially the MKK, Sandawe and Ethiopian Jews, can form a distinct grouping (pink), in which case other Tanzanian populations, the Maasai and Chagga, and the Somali (now living in Pakistan) appear intermediate between East and West Africa. The next consistent cluster includes the Mozabites and Southwest Asians (green). There is then a more-or-less gradient across Europe from southeastern and southern Europe (mostly green), through to northwestern Europe, and ending in northeastern Europe (mostly yellow). The south-central Asian populations form another (dark blue) consistent and relatively homogeneous cluster of populations, including East Indians and several Pakistani populations (dark blue). The Khazaks, Uyghur, Hazara and Khanty form a "group" that is depicted as admixed under any of the alternative common patterns. The next group of populations (dark gray) appears homogeneous from the Khamba-Tibet through Southeast Asia all the way to East Asia but the alternative (pattern B in Figure 4) has the western Chinese groups at one end of a more clinal pattern with the southeastern Asians at the other end. Interestingly, this alternative depicts the Han, Koreans and Japanese as admixed. The next clear cluster (light blue) is Pacific and consists of three Melanesian samples: The Samoans, Micronesians and Malaysians appear intermediate between East Asia and the Pacific. The final clear cluster (pink) consists of Native American samples.

**Figure 5:**
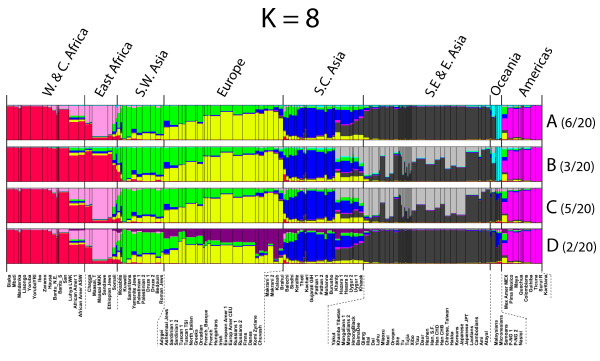
**Average population assignment to clusters for *structure *analyses at *K *= 8**. The data are the same as the *K *= 8 analysis in Figures 3 and 4.

The different patterns at *K *= 7 and *K *= 8 show fine distinctions even among the regions that are superficially similar. To make some of these clearer, we have generated the population averages for the best result (highest likelihood) for each of the patterns (Figure 5). These emphasize the variation among individuals in each population sample by showing the population as multiple colors. These figures also emphasize the southwestern Asia through northern Europe cline seen in all patterns.

Clusters that emerge at even higher values of *K *include a Pygmy/San/S Bantu cluster in Africa, a Khanty/Khazak/Yakut cluster in Asia and a vaguely central Asian group consisting of, for example, the Khamba-Tibet, Mongolian, Baima Dee and Qiang. These clusters, though reasonable, are not strongly supported statistically.

## Discussion

A set of markers particularly useful for determining in detail the genetic distinctions among populations should also be useful in an examination of admixture. However, "admixture" is not a singular phenomenon: A sample of individuals might be considered admixed if it is composed of (1) samples from two or more different populations, (2) the descendants of people from two or more populations who have "recently" intermarried, (3) descendants of people from two or more populations who have intermarried in the ancient past and (4) people discretely sampled from a single region along a geographic allele frequency cline established predominantly by random genetic drift.

The American Society of Human Genetics Ancestry and Ancestry Testing Task Force, in its white paper [40], sets forth caveats to be kept in mind in ancestry inference, perhaps the foremost of which is that ancestral populations cannot be observed directly and that even surrogates for those ancestral populations may not be included in any given study. Therefore, the "gold standard" analytic programs such as *structure *(version 2.3.3; http://pritch.bsd.uchicago.edu/structure.html[32-34]) will cause individuals in some populations to appear as an "admixture" of the population samples that are in the analysis. Even in analyses of principal components, it is not possible to distinguish whether a population is admixed or simply intermediate. Thus, a set of AIMs estimating the ancestry of an individual whose ancestry involves populations other than the majority of the populations in an analysis may be unsatisfactory by forcing that individual to be explained by the ancestry inferred for the majority. Further, a set of AIMs selected for one set of populations cannot be expected to be as good at distinguishing among other populations, perhaps even from the same geographic regions.

It is important to realize that the outcome of any analysis of admixture or other population structure depends heavily on both the population samples and the markers used. Though we have included some of the same HGDP populations as Kosoy *et al*. [6] did in their analyses, the outcome is always a function of which samples are included. Thus, in our selection of samples, we have also included samples that overlap with those reported by Nassir *et al*. [27] as well as others not part of either the Kosoy *et al*. [6] or Nassir *et al*. [27] reports.

### PCA

The results shown in Figure 2 clearly reflect the criteria used to select this set of AISNPs [6]. The strongest discrimination reflects the geographic and ancestral origins of those populations (Africa, Europe, East Asia and the Americas), even though this analysis included none of the original samples used to select the SNPs. The first two components provide strong support for these SNPs in studies involving African, European and Native American populations. The relatively poorer separation among Eurasian and Pacific populations reflects the absence of Central, South and East Asian and Pacific populations in the selection of these AIMs as well as their distinct evolutionary relationships relative to African and Native American populations. It is logical to expect that if more SNPs with large allele frequency differences across Eurasia were included, factor 3 would show greater separation between west and east Eurasia.

#### Structure

*Structure *attempts to find the set of K population allele frequencies that will give the best fit to all individual samples assuming Hardy-Weinberg ratios for each of the K populations. *Structure *does not consider or produce analyses of population relationships. Fortunately, this is not an issue of interest to forensic science. Rather, *structure *assigns individuals to clusters of genetically similar individuals. Obviously, if numbers of individuals differ greatly among different populations, a population sample with a large number of individuals will influence the allele frequencies of the particular cluster into which it falls more than a population with a small number of individuals. Thus, a small population from the middle of a cline with larger numbers in populations from the more extreme parts of the cline will appear "admixed." Such is seen at *K *= 3 for the South, Central and East Asian populations. However, a large population from that middle region will, at the same *K *value, cause the allele frequency estimates of the flanking clusters to move toward the center even if cluster assignments do not change. The consequences at higher K values may be that the "middle" population is a distinct group or, by shifting the estimates for the flanking clusters, cause a population at the extreme of the cline to "fall off" and become a separate cluster. The cluster assignments at *K *= 4 and *K *= 5 illustrate this (Figure 3). In other words, conclusions about groupings at a given value of *K *are a function of the populations sampled and their relative sample sizes. Thus, it is not necessarily correct that the estimated allele frequencies for a given cluster represent the ancestral population, nor can one automatically interpret a partial assignment to two or more clusters as admixture. In addition, as shown in Figure 4 and Table 2, there is a stochastic element in each *structure *run such that the relative likelihoods of different patterns from different runs depend on the particular outcomes that happen to occur. Thus, the point of using *structure *is not the single best run or the most common pattern seen, but the stability of aspects of the patterns and of the individual runs within each pattern among the runs with the higher likelihoods. Kalinowski [41] has recently published studies making additional relevant points on the interpretation of *structure *results.

An example of the sample size effect appears to be found in Nassir *et al*. [27]. That study contains 49 populations with a total sample size of 1,620. Their Ashkenazi sample of 240 individuals (two population samples pooled: Ashkenazi AM 4 GP and Ashkenazi AM) constitutes about 15% of the total. Similarly, the 259 European Americans (two population samples pooled: European AM CEU and European AM NYCP) constitute about 16% of the total. These two heavily weighted population samples probably decrease the resolution of European and southwestern Asian populations. Our data set with the same sites and no population consisting of more than 6% (Han, pooling four population samples, CHB, CHD, SF and Taiwan = 5.4%) of the total sample can begin to distinguish a southwestern Asian cluster at *K *= 6, though showing a cline through Europe. Unfortunately, almost all of our East Asian samples, including many Chinese minorities, are *de facto *similar, with this set of AISNPs constituting the equivalent of nearly a quarter of our whole sample through *K *= 8, clearly affecting how South Asian and especially Central Asian populations appear. There are, however, differences among them sufficient to result in a more complex clinal pattern as a reasonable alternative at *K *= 7 and *K *= 8 (Figure 4). In the ideal world, a world we doubt exists, all samples would be large, equal in size and evenly distributed around the world.

### Forensic Implications

Our analyses have been directed toward evaluating this set of SNPs for a particular purpose: ancestry inference as an investigatory tool. We have used PCA and *structure *for these evaluations. However, we do not advocate using either PCA or *structure *as a forensic tool for inference of individual ancestry in casework. Direct evaluation by likelihood methods is much more accurate. Any polymorphism can also be used to assist in matching crime scene and suspect DNA genotypes and to estimate the probability of the match occurring by chance if allele frequency data exist. Therefore, these 128 AISNPs could be used for exclusion, but we would not advise use of these markers to estimate the probability of a match occurring by chance. They have been selected to distinguish among populations and to have highly varying frequencies. To use these data in a court, one would have to present a diverse set of calculations and assumptions. The complexities of the calculations and the assumptions would allow an easy challenge, and all potential benefits of SNPs over the standard CODIS markers would be lost. There are good panels of SNPs selected for individual identification [e.g., [25,26]. The set of SNPs for individual identification that we developed [26] largely circumvents the problem of different allele frequencies in populations from different parts of the world. Similarly, we feel the 128 AISNPs analyzed in this paper are not efficient for any estimates of phenotype beyond the very indirect inference from ancestry.

The data for these SNPs can be used to "assign" regional ancestry to a single individual based on the genotypes at all or a significant fraction of these 128 SNPs. This would be done by calculating the likelihood of the multisite genotype based on the allele frequencies of each of the 119 population samples (frequencies are in ALFRED [37]). It is clear that for many genotypes, many populations will have roughly comparable likelihoods. The clusters at *K *= 9-11 (not shown) indicate no new strongly supported subgroups of populations and suggest, for example, that differentiating ancestry from among populations within East Asia will not be easy using the allele frequencies for this set of SNPs.

It is important to distinguish population averages from the variation among individuals (Additional files 6 and 7) within that population. Figure 5 presents the population averages for the *K *= 8 structure analysis. Compared to the variation among individuals shown in Figure 4, the averages make some of the global patterns clearer but completely obscure the individual variation that can be of great importance in a forensic setting.

In a comparative examination of a total of seven small publicized AISNP panels containing a total of 688 SNPs, we found that only one SNP (rs2065160) occurred in three of the panels and 26 other SNPs (about 4%) occurred in two panels. None of the 128 SNPs in the panel we have analyzed occurred in any of these other panels. The small number of overlapping SNPs across panels likely results from the different methods of selecting SNPs, the different data sets from which SNPs are selected and the different purposes of the panels: some are global, some are regional and some are for the four continental extremes. However, though the specifics of these panels are not relevant, it is clear that there is no single set of AISNPs that will be of value for all questions.

With our additional data and the analyses presented here, this panel of 128 AISNPs is the best documented and validated for broad global application to infer ancestry. However, it is not necessarily the optimal panel depending on the question being asked, and it is definitely not optimal at identifying ancestry within Europe and Southwest Asia (cf. Figures 3, 4 and 5; *K *= 6-8). Distinguishing among East Asian populations is also not optimal with this set of AISNPs. Neither of these conclusions is surprising, since populations in those regions were not part of the selection of this set of AISNPs. Selection to identify SNPs with markedly different allele frequencies across East Asia will be necessary [24]. Many useful SNPs must already exist; the problem is to identify them. In general, as more and more SNPs are identified through ongoing sequencing projects, other SNPs may be optimal for resolving population similarities within one of the major clusters in the *structure *analyses of Figures 3 and 4. However, comparison of the relative discriminating ability of additional candidate SNPs requires that all SNPs be typed on the same populations and, ideally, the same individuals. That will require coordination among laboratories and sharing of data and/or samples. We have put all of the allele frequencies of the populations we have studied in this paper into ALFRED [30]; the raw individual genotype data are available on request.

## Competing interests

The authors declare that they have no competing interests. FMDLV is employed by Life Technologies.

## Authors' contributions

All authors have read and approved the final manuscript. KKK and FMDLV were involved in the conception and design of the study, and KKK assisted in writing the manuscript. JRK supervised the genotyping assays and data analysis and wrote the manuscript. FRF and AJP assisted in the data analysis. WCS assembled and integrated the data set from the literature and laboratory.

## Supplementary Material

Additional file 1 **List of missing values and how they were handled in the PCA**. Click here for file

Additional file 2 **Population allele frequencies**. Click here for file

Additional file 3 **List of Fst values for all 128 AISNPs**. Click here for file

Additional file 4 **List of upper and lower outliers for Fst in Reference and AISNP distributions**. Click here for file

Additional file 5 **Likelihood plot K = 2-12**. Click here for file

Additional file 6 **Individual Assignments in *Structure *analysis**. Click here for file

Additional file 7 **Population Assignments in *Structure *analysis**. Click here for file
