# Supplementary material for: Analyses of a set of 128 ancestry informative single-nucleotide polymorphisms in a global set of 119 population samples
Source: Investig Genet. 2011 Jan 5;2:1. doi: 10.1186/2041-2223-2-1 (PMC3025953; doi:10.1186/2041-2223-2-1)
Supplement: Additional file 1 — List of missing values and how they were handled in the PCA. [file 2041-2223-2-1-S1.DOC]

Additional File 1.

Instances of missing data.

As can be seen these occurred exclusively in the HapMap samples but are expected to have no effect on the analyses. For PCA the average allele frequencies of the otherwise most similar populations was substituted to generate a complete matrix of values.

| Population | Missing AISNP Typing |
| --- | --- |
| CHD | rs10108270 |
| CHD | rs12130799 |
| LWK | rs12130799 |
| LWK | rs12439433 |
| MKK | rs12439433 |
| LWK | rs3907047 |
| MKK | rs3907047 |
| MEX | rs4670767 |
| MEX | rs4800105 |
| LWK | rs5768007 |
| TSI | rs6541030 |
| GIH | rs6541030 |
| LWK | rs8113143 |
| LWK | rs818386 |
| ASW | rs10513300 |
| ASW | rs12439433 |
