# Supplementary material for: Analyses of a set of 128 ancestry informative single-nucleotide polymorphisms in a global set of 119 population samples
Source: Investig Genet. 2011 Jan 5;2:1. doi: 10.1186/2041-2223-2-1 (PMC3025953; doi:10.1186/2041-2223-2-1)
Supplement: Additional file 4 — List of upper and lower outliers for Fst in Reference and AISNP distributions. [file 2041-2223-2-1-S4.DOC]

Additional File 4A.

Outliers in the two Fst distributions.

Outlier SNPs at the upper ends and the Lower ends of each of the two Fst distributions

in Figure 1.

| **ANCESTRY INFORMATIVE SNPS** | | | | | |
| --- | --- | --- | --- | --- | --- |
| **Upper Outliers** | | | **Lower Outliers** | | |
| **Locus** | **RS#** | **Fst** | **Locus** | **RS#** | **Fst** |
| *EDAR* | rs260690 | 0.52 | *TWSG1* | rs4798812 | 0.08753 |
| *RTTN* | rs4891825 | 0.52 |  |  |  |

Additional Table 4B. Outliers

| **REFERENCE SNPS** | | | | | |
| --- | --- | --- | --- | --- | --- |
| **Upper Outliers** | | | **Lower Outliers** | | |
| **Locus** | **RS#** | **Fst** | **Locus** | **RS#** | **Fst** |
| *SLC24A5* | rs1426654 | 0.77 | *CCDC8* | rs414974 | 0.04 |
| *OCA2* | rs1800414 | 0.59 | *DLX4* | rs486993 | 0.04 |
| *OCA2* | rs1129038 | 0.55 | *ABACC3* | rs12051822 | 0.04 |
| *HERC2* | rs12913832 | 0.55 | *GOSR2* | rs9898527 | 0.04 |
| *EDAR* | rs260690 | 0.54 | *CYP2C9* | rs1057911 | 0.04 |
| *CCR7* | rs3136687 | 0.54 | *TRIM34* | rs7947715 | 0.03 |
| *SORCS3* | rs7914674 | 0.52 | *SLC35B1* | rs1745294 | 0.03 |
|  |  |  | *JAZF1* | rs849140 | 0.03 |
|  |  |  | *CD4* | rs35259686 | 0.02 |
|  |  |  | *MC1R* | NA | 0.02 |
