# Supplementary material for: Analyses of a set of 128 ancestry informative single-nucleotide polymorphisms in a global set of 119 population samples
Source: Investig Genet. 2011 Jan 5;2:1. doi: 10.1186/2041-2223-2-1 (PMC3025953; doi:10.1186/2041-2223-2-1)
Supplement: Additional file 5 — Likelihood plot K = 2-12. [file 2041-2223-2-1-S5.PDF]

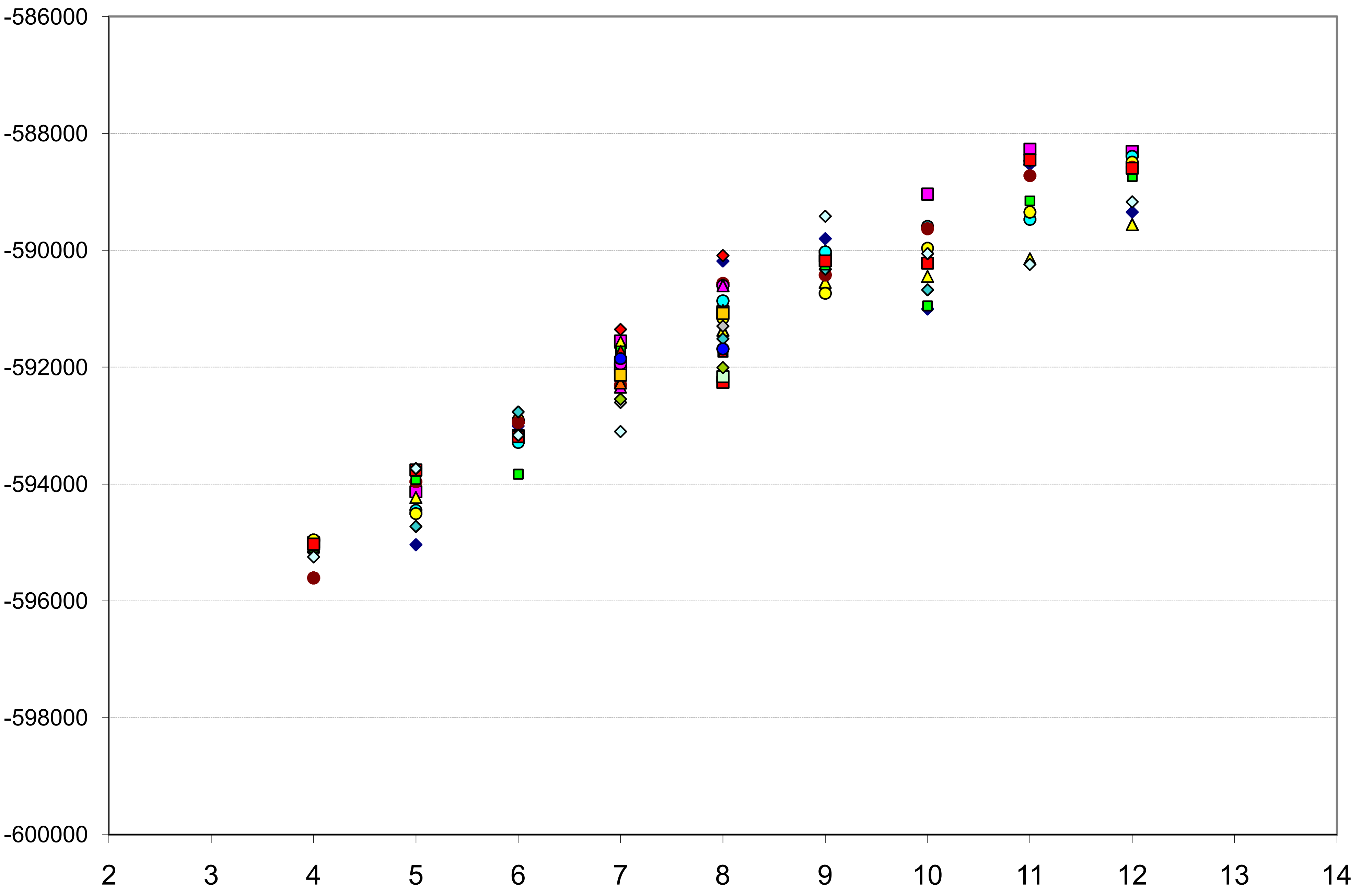

Additional File 5. Likelihood plot for K=4-12. Likelihood values for each structure run, K=2 through K=12. There are 10 replicates for K=2 to K=6 and K=9 to K=12. At the K values of 7 and 8 there are 20 replicates. (The likelihood values for K=2 and 3 are low and off the scale.) Although the highest likelihood values at K=9-11 continue to increase, they do so at a decreasing rate and we chose K=8 as a conservative stopping point.
